# Supplementary material for: Aggregation is a Context-Dependent Constraint on Protein Evolution
Source: Front Mol Biosci. 2021 Jun 18;8:678115. doi: 10.3389/fmolb.2021.678115 (PMC8249573; doi:10.3389/fmolb.2021.678115)
Supplement: Supplementary file 1 [file Presentation1.pdf]

Aggregation is a Context-Dependent Constraint on Protein Evolution

Michele Monti*,[1,2] Alexandros Armaos,[3,2] Marco Fantini,[4] Annalisa Pastore,[5] and Gian Gaetano Tartaglia*[6,2,7]

[1]*Centre for Genomic Regulation (CRG), The Barcelona Institute for Science and Technology, Barcelona, Spain*
[2]*RNA System Biology Lab, Centre for Human Technologies, Istituto Italiano di Tecnologia (IIT), Genoa, Italy*
[3]*Centre for Genomic Regulation (CRG), The Barcelona Institute for Science and Technology, Barcelona, Spaim*
[4]*Department of Chemistry, Columbia University, New York, NY 10027, USA*
[5]*3UK-DRI Centre at the Maurice Wohl Institute, King's College London, London, UK*
[6]*Centre for Genomic Regulation (CRG) and ICREA, The Barcelona Institute for Science and Technology, Barcelona, Spain*
[7]*Sapienza University, Dipartimento di Biologia e Biotecnologie, Rome, Italy*

I. HUMAN PROTEOME

In Figure 1 we report (A) the distribution of sequences length and (B) the frequencies of amino acids in the human proteome. The histograms in Figure 2 show four curves, two for the aggregation and two for the folding propensities, computed with both uniformly and weighted distributed amino acids (distribution shown in Figure 1 right panel). We found that the folding propensity is reduced through the process of randomization while the aggregation increase on average.

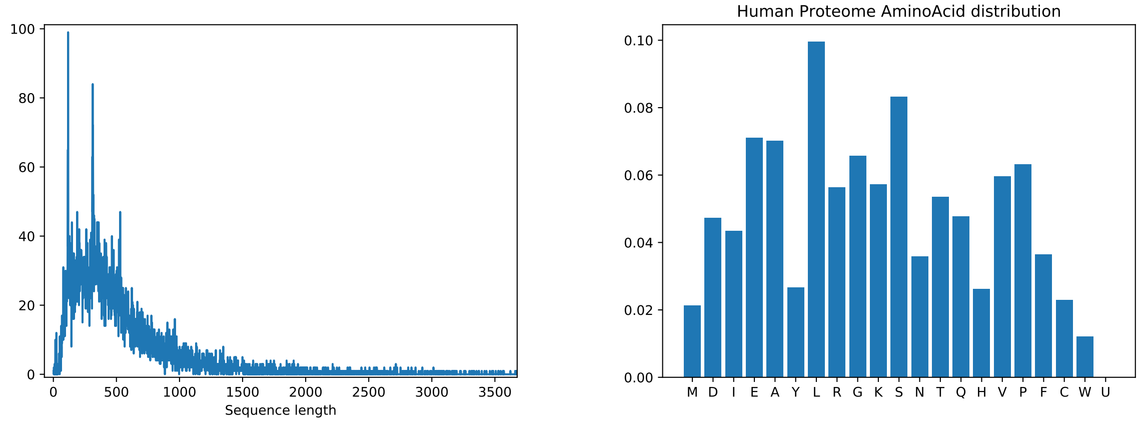

FIG. 1: Statistical analysis of the human proteome. On the left we report the distribution of the lengths of protein sequences and on the right the amino acid frequencies.

II. TDP-43 EXPRESSION IN S. CEREVISIAE

Analysis carried out with the Clever Machine approach reveals that aggregation strongly discriminates high vs low fitness mutants (5000+5000 instances analysed):

`http://crg-webservice.s3.amazonaws.com/submissions/2020-09/295992/output/index.html?unlock=e24ba9a2ff`.

For different high vs low fitness scores, we report the performances of the linearized Zyggregator algorithm computed using the Area under the Receiver Operating Characteristics Curve (AUC of ROC; Figure 3). In Figure 4 we show

* Corresponding authors: michele.monti@iit.it, Annalisa.Pastore@crick.ac.uk, gian.tartaglia@iit.it

the predicted aggregation propensities for the experimental negative set and its random version (single and double mutations).

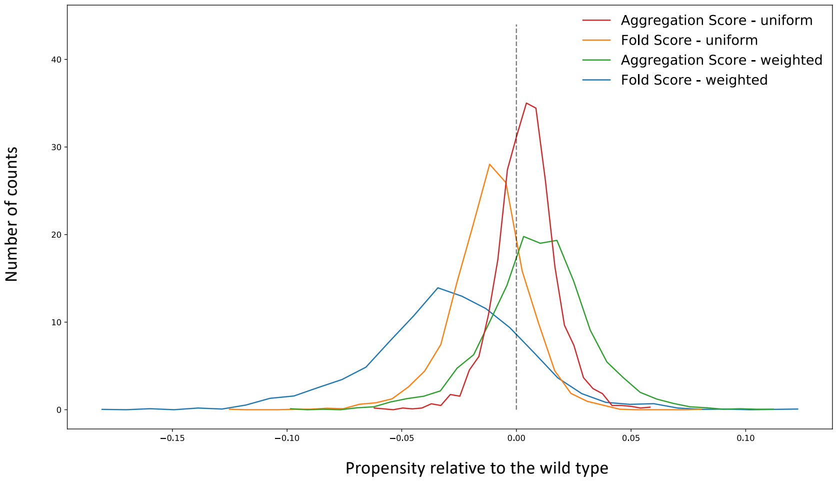

FIG. 2: Difference between aggregation and folding propensities of protein variants. Each protein underwent 500 random mutations respect to the WT one. The histograms are computed over 10 000 human proteins. The random is computed in two ways: considering a uniform amino acid distribution or using the human proteome amino-acid occurrences. The results do not change.

III. TEM BETA-LACTAMASE EXPRESSION IN E. COLI

AUC for all the sub datasets regarding the TEM Beta-Lactamase experiment. In Figure 5 we show how we filtered the data taking into account only the AUC that have been computed for significantly populated datasets. Datasets of a small size and with an higher number of mutations respect to the mean of the generation class are discarded. Sequences with higher number of mutations are more prone to have a lower fitness propensity, as shown in Figure 5.

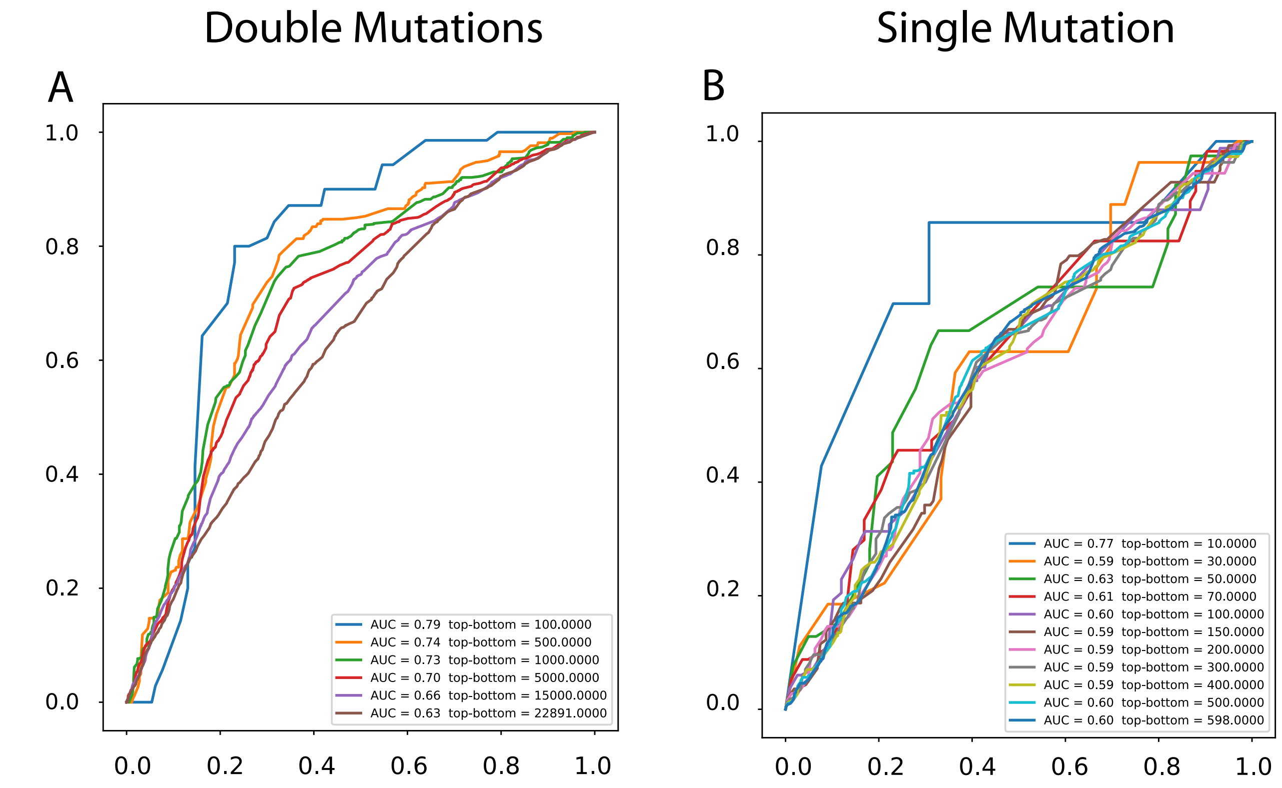

FIG. 3: Aggregation propensity of TDP-43 mutants discriminate high vs low fitness scores. The ROC curves were computed for different high vs low fitness mutations. Panel A reports on the double mutations dataset and panel B informs about single mutations .

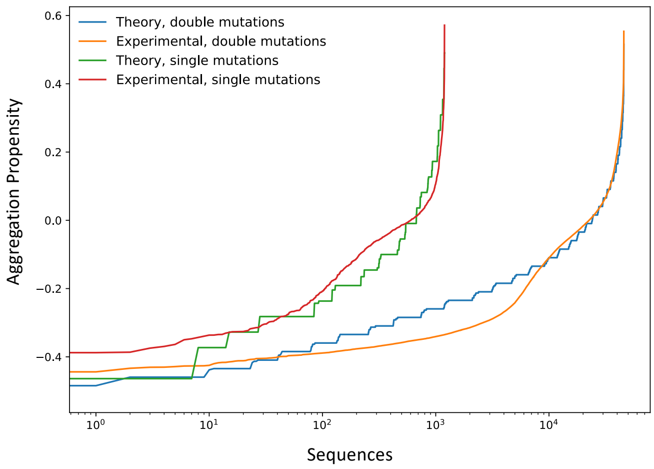

FIG. 4: Aggregation propensities of experimental and theoretical mutants and related aggregation propensities.

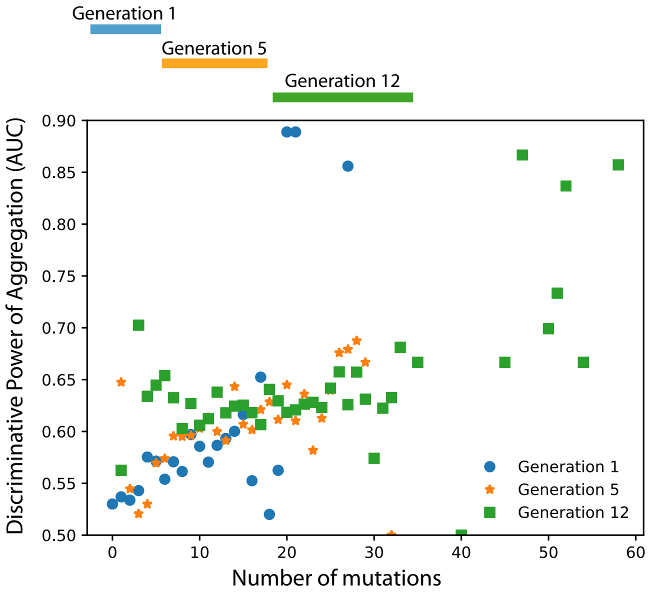

FIG. 5: AUC computed for TEM Beta-Lactamase mutants. In the analysis we selected only points belonging to datasets of significant size (mutational range highlighted with colored bars; Materials and Methods).
